# Bacurd2 is a novel interacting partner to Rnd2 which controls radial migration within the developing mammalian cerebral cortex

**DOI:** 10.1186/s13064-015-0032-z

**Published:** 2015-03-31

**Authors:** Ivan Enghian Gladwyn-Ng, Shan Shan Li, Zhengdong Qu, John Michael Davis, Linh Ngo, Matilda Haas, Jeffrey Singer, Julian Ik-Tsen Heng

**Affiliations:** EMBL Australia, The Australian Regenerative Medicine Institute, Monash University, Clayton, Victoria 3800 Australia; Department of Biology, Portland State University, Portland, Oregon 96207 USA; The Harry Perkins Institute of Medical Research, Perth, Australia; Centre for Medical Research, The University of Western Australia, Perth, Australia; Present address: The Harry Perkins Institute of Medical Research, Perth, Australia

**Keywords:** Neuronal migration, Cerebral cortex, Rho GTPase, Bacurd2, Tnfaip1, Rnd2

## Abstract

**Background:**

During fetal brain development in mammals, newborn neurons undergo cell migration to reach their appropriate positions and form functional circuits. We previously reported that the atypical RhoA GTPase Rnd2 promotes the radial migration of mouse cerebral cortical neurons (Nature 455(7209):114–8, 2008; Neuron 69(6):1069–84, 2011), but its downstream signalling pathway is not well understood.

**Results:**

We have identified BTB-domain containing adaptor for Cul3-mediated RhoA degradation 2 (Bacurd2) as a novel interacting partner to Rnd2, which promotes radial migration within the developing cerebral cortex. We find that Bacurd2 binds Rnd2 at its C-terminus, and this interaction is critical to its cell migration function. We show that forced expression or knockdown of *Bacurd2* impairs neuronal migration within the embryonic cortex and alters the morphology of immature neurons. Our *in vivo* cellular analysis reveals that *Bacurd2* influences the multipolar-to-bipolar transition of radially migrating neurons in a cell autonomous fashion. When we addressed the potential signalling relationship between Bacurd2 and Rnd2 using a Bacurd2-Rnd2 chimeric construct, our results suggest that Bacurd2 and Rnd2 could interact to promote radial migration within the embryonic cortex.

**Conclusions:**

Our studies demonstrate that *Bacurd2* is a novel player in neuronal development and influences radial migration within the embryonic cerebral cortex.

**Electronic supplementary material:**

The online version of this article (doi:10.1186/s13064-015-0032-z) contains supplementary material, which is available to authorized users.

## Background

During mammalian brain development, newborn neurons undergo a well-defined migratory journey in order to arrive at their final location within the developing nervous system and form functional connections with other neural cells [1-3]. Following their birth within the germinal zone of the ventricular neuroepithelium (known as the ventricular zone (VZ)), they migrate through a transitional intermediate zone (IZ) before arriving at their appropriate positions within the cortical plate (CP) and undergo terminal differentiation. Failure in the proper positioning of neurons during brain development can result in the formation of abnormal neural circuits, leading to intellectual impairment and epilepsy in humans [4,5].

While the molecular mechanisms which govern cell migration during brain development are not fully understood, recent work has revealed that neuronal migration is intrinsically regulated by the activity of DNA binding transcription factors on a RhoA-like GTPase gene known as *Rnd2* [6,7]. It was discovered that members of the basic helix-loop-helix (bHLH) family of transcriptional activators (such as Neurog2, NeuroD1 and NeuroD2) stimulate *Rnd2* expression to promote the migration of newborn excitatory neurons of the cerebral cortex [6,8]. Furthermore, transcriptional repressors such as COUP-TFI and RP58 negatively regulate *Rnd2* expression in the course of their radial migration and control their multipolar-to-bipolar conversion within the IZ as they enter the CP to complete their migration [9-11]. Together, these multiple regulatory pathways control appropriate levels of *Rnd2* gene dosage in neurons to shape their development during cortical neurogenesis.

Despite a deep understanding of the regulation of *Rnd2* expression for the positioning of neurons within the nascent cortex, the intracellular signalling pathways through which Rnd2 controls cell migration remain less well understood. Nevertheless, Rnd2 and its related family member Rnd3 are both known to control radial migration and neurite outgrowth through their actions on the actin cytoskeleton [6,7,12]. However, while recent studies demonstrate that both Rnd proteins commonly suppress RhoA signalling and modulate the filamentous-actin (F-actin) cytoskeleton within cortical neurons as they differentiate within the embryonic cortex [7], the underlying signalling mechanisms for Rnd2 and Rnd3 are known to be different. Notably, Rnd3 mediates actin depolymerisation and promotes cell migration within the embryonic cortex through its downstream effector molecule p190RhoGAP, while Rnd2 does not signal through this pathway [7]. In addition, Rnd proteins are known to interact with different protein partners in order to elicit their effects on fibroblast cell shape and motility (reviewed in [13,14]), thus the challenge remains to better understand the complexity of the downstream signalling pathways through which Rnds function in neural cells as well.

In this study, we wanted to clarify the signalling pathway through which Rnd2 mediates cell migration during neuronal development in mice. We have identified a member of the BTB-domain containing adaptor for Cul3-mediated RhoA degradation (Bacurd2) as a novel binding partner to Rnd2 within the mouse embryonic cerebral cortex. We report that knockdown or forced expression of *Bacurd2* disrupts radial cell migration *in vivo* and that Bacurd2 promotes the multipolar-to-bipolar transition of neurons as they transit from the intermediate zone into the cortical plate. In our exploration of the functions for Bacurd2 and Rnd2, we find both to be crucial to the migration of newborn neurons within the embryonic cerebral cortex.

## Results

### Bacurd2 interacts with Rnd2 and mediates cell migration within the embryonic cerebral cortex

**Figure 1:**
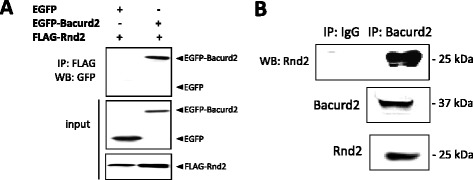
**Bacurd2 interacts with Rnd2** ***in vitro*** **and** ***in vivo*** **. (A)** Immunoprecipitation assays with transiently transfected HEK293T cells show that FLAG-Rnd2 interacts with EGFP-Bacurd2, but not EGFP. **(B)** Lysates of E14.5 mouse brain homogenates were immunoprecipitated with a Bacurd2 antibody and probed by immunoblotting for Rnd2 to confirm their interaction *in vivo*. As a control, mouse-anti-IgG did not immunoprecipitate Rnd2. Input lanes confirm protein expression in both experiments. Further details of antibodies used to detect Bacurd2 and Rnd2 are provided in Additional file 2: Figure S2.

To identify binding partners to Rnd2, we performed a yeast two-hybrid screen of an embryonic mouse (E15.5) cortex library [15] using an Rnd2 bait construct lacking the C-terminal membrane-binding (CAAX) motif. A survey of 2 × 10^7^ independent clones resulted in the isolation of multiple interacting prey clones encoding polypeptides corresponding to full-length Bacurd2, as well as a smaller fragment comprising the C-terminal aa242-316 fragment. Following prey plasmid recovery, complementation tests confirm specificity of interaction between Bacurd2 preys and the Rnd2 bait, but not pLaminC or with p53 (Additional file 1: Figure S1). To confirm protein-protein interaction between Bacurd2 and Rnd2, we performed immunoprecipitation experiments with epitope-tagged constructs and found that FLAG-tagged Rnd2 binds to EGFP-Bacurd2 fusion protein, but not to EGFP alone (Figure 1A). We also performed immunoprecipitation experiments with mouse embryonic (E14.5) brain lysate using a Bacurd2 antibody (Additional file 2: Figure S2A) to confirm their interaction *in vivo* (Figure 1B). Bacurd2 and Rnd2 are detected throughout the course of brain development (Additional file 2: Figure S2C). Immunostaining of embryonic E14.5 cerebral cortex tissue revealed Bacurd2 signal in the VZ, sVZ and IZ, while parallel experiments performed with pre-immune serum did not elicit a signal (Additional file 2: Figure S2D-E).

**Figure 2:**
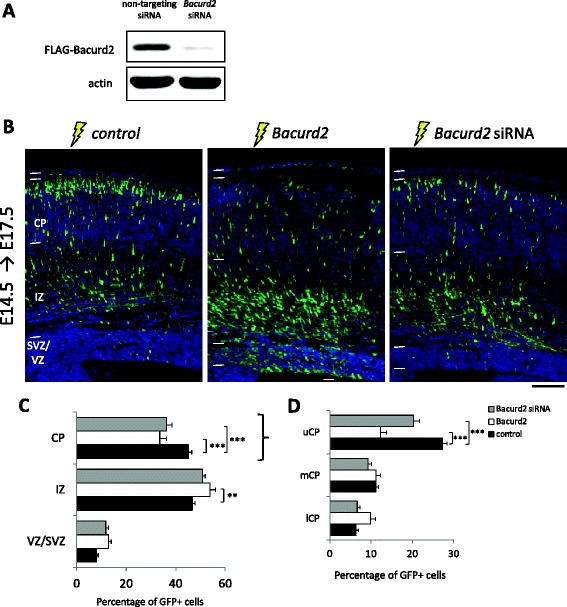
**Bacurd2 influences cell migration within the embryonic mouse cerebral cortex. (A)** Western blotting with HEK293T cell lysates confirms that FLAG-Bacurd2 expression is suppressed by targeting siRNAs, but not by control (non-targeting) siRNAs. Actin was used as loading control. **(B)** *In utero* electroporation was performed on embryonic mouse E14.5 embryos and analysed 3 days later at E17.5. Cortical cells were electroporated with control vector (GFP only), a bicistronic GFP expression construct which also encodes Bacurd2, or *Bacurd2* siRNA co-electroporated with GFP vector. **(C)** Quantification reveals that forced expression of Bacurd2, or treatment with *Bacurd2* siRNAs, alters the distribution of cells within the embryonic cortex (*N* > 4,500 cells from four to six brains per condition; *F*_4,72_ = 14.97; *P* < 0.0001; two-way ANOVA followed by Bonferroni’s *post hoc* test; ****P < 0.0001. **(D)** Quantification of GFP+ cells within the CP (divided into the lower, medial and upper CP) reveals that forced expression of Bacurd2 or knockdown of Bacurd2 disrupts the intracortical distribution of GFP+ cells compared with control (*N* > 1,500 cells from four to six brains per condition; *F*_4,72_ = 27.89; *P* < 0.0001; two-way ANOVA followed by Bonferroni’s *post hoc* test). uCP, mCP and lCP indicate upper, medial and lower cortical plates, respectively. Scale bar represents 100 μm.

Next, we performed a series of *in utero* electroporation experiments on E14.5 mouse embryonic cortex to determine whether perturbations to *Bacurd2* might disrupt cortical development. To do this, we forced expressed *Bacurd2* by delivering a bicistronic expression construct encoding Bacurd2 and GFP into embryonic cortical cells and examined the distribution of GFP-labelled cells 3 days later at E17.5. In a reciprocal approach, we suppressed *Bacurd2* expression in cells using targeting siRNAs together with an empty (GFP only) vector (Figure 2A). In each condition, the amounts of siRNA (control or targeting) and expression vector (GFP only, or GFP + Bacurd2 bicistronic vector) were normalised to enable comparisons across conditions. In Figure 2B, we show that while a significant proportion of GFP-labelled cells had migrated into the CP of control-treated brains, forced expression of *Bacurd2* or knockdown with siRNAs disrupted their migration within the embryonic cortex, observed as an accumulation of cells within the IZ and a concomitant decrease in cells located within the CP (Figure 2C). Within the CP, a significant proportion of *Bacurd2*-overexpressing cells and *Bacurd2* siRNA-treated cells failed to reach the upper cortical plate, suggesting that changes to *Bacurd2* levels disrupt their ‘intracortical’ positioning (Figure 2D). To account for the possibility that disruptions to *Bacurd2* might influence cortical neurogenesis, we performed quantification studies and found no significant differences in the proportions of GFP+/Tuj1+ cells or their distribution within the subcompartments of the embryonic E17.5 cortex (Additional file 3: Figure S3).

**Figure 3:**
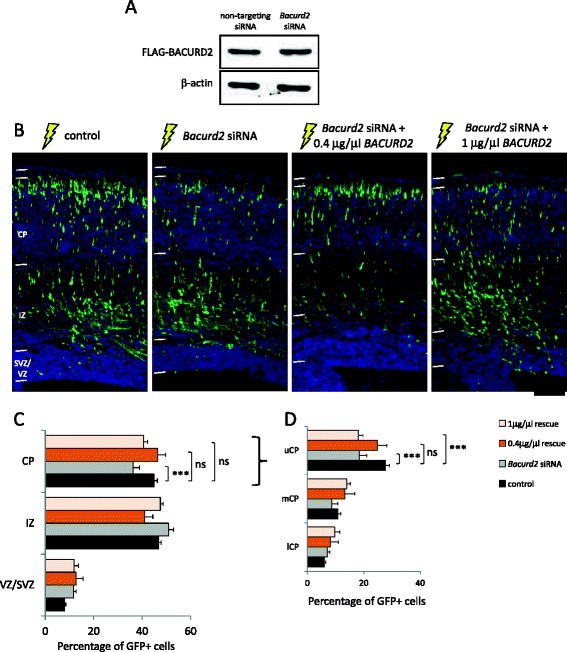
**The defective migration of** ***Bacurd2*** **siRNA-treated cells is augmented by co-delivery of human BACURD2. (A)** Western blotting of lysates from P19 embryocarcinoma cells transiently transfected with control siRNA or *Bacurd2* siRNAs, together with an expression construct encoding human BACURD2 as an epitopte-tagged (FLAG) protein. FLAG-BACURD2 protein expression is refractory to Bacurd2 siRNA-mediated knockdown. **(B)** *In utero* electroporation studies with E14 mouse brains electroporated with GFP vector and control siRNA (‘control’), GFP vector and *Bacurd2* siRNA, and *Bacurd2* siRNA with the indicated concentrations of BACURD2 expression construct are indicated. **(C)** Quantitation reveals that while Bacurd2 siRNA treatment impairs radial migration, co-delivery of 0.4 μg/μl BACURD2 construct restores their migration to control levels, while co-delivery of 1.0 μg/μl BACURD2 construct only partially restores their migration within the embryonic cortex (*N* > 1,450 cells counted per condition; *F*_6,45_ = 15; *P* < 0.0001; two-way ANOVA followed by Bonferroni’s *post hoc* test). **(D)** An analysis of their intracortical distribution reveals that the defective migration of *Bacurd2* siRNA-treated cells is restored with co-delivery of 0.4 μg/μl of BACURD2 construct (*N* > 500 cells per condition; *F*_6,42_ = 15; *P* < 0.0001; two-way ANOVA followed by Bonferroni’s *post hoc* test). Graph plots mean ± SEM. Scale bar represents 100 μm.

To confirm the specificity of the siRNA-mediated migration defect, we performed rescue experiments whereby cells were co-treated with an expression construct encoding human BACURD2 which was refractory to RNAi (Figure 3). Our results show that the defective migration of siRNA-treated cells could be significantly restored to levels resembling control condition when 0.4 μg/μl of BACURD2 construct was co-delivered with *Bacurd2* siRNA (Figure 3C). Interestingly, while co-treatment with either concentrations of BACURD2 enhanced migration into the CP (Additional file 4: Figure S4), we show in Figure 3C,D that the migration profile of siRNA-treated cells was corrected to levels resembling control when co-treated with 0.4 μg/μl of BACURD2 (Figure 3C), while co-treatment with a higher concentration (1 μg/μl) of BACURD2 construct disrupted intracortical positioning (Figure 3D). Thus, Bacurd2 cell autonomously controls radial migration, with concentration-sensitive effects.

**Figure 4:**
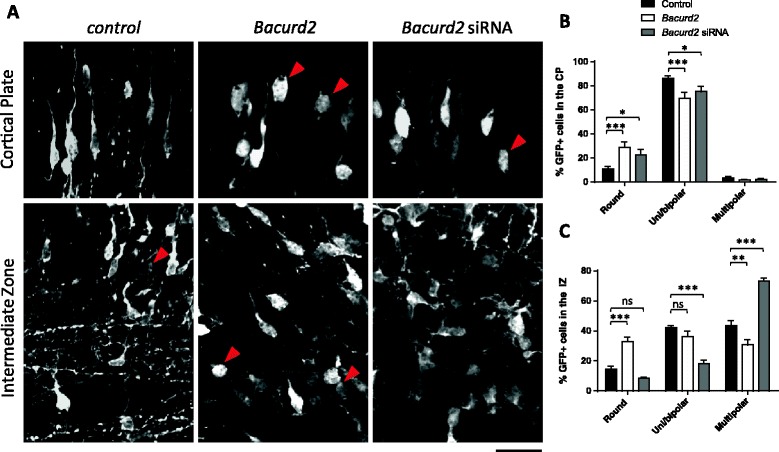
**The effect of forced expression or knockdown of Bacurd2 on the morphology of GFP-labelled neurons within the IZ and CP of the E17.5 embryonic cortex. (A)** The morphology of neurons within the CP and the IZ in representative brain sections electroporated with control (GFP only) vector, *Bacurd2* expression vector or *Bacurd2* siRNAs. Arrowheads point to round-shaped cells. **(B)** Within the CP, overexpression or knockdown of Bacurd2 leads to a significant increase in the proportion of round cells, and a decrease in uni/bipolar-shaped cells (*N* > 300 cells counted from three brains per condition; *F*_4,45_ = 9.63; *P* < 0.0001, two-way ANOVA followed by Bonferroni’s *post hoc* test; *P < 0.05, ***P < 0.001). **(C)** Within the IZ, overexpression of *Bacurd2* leads to a significant increase in the proportion of round cells and a decrease in multipolar-shaped cells (*N* > 500 cells counted from three brains per condition; *F*_4,33_ = 48.56; *P* < 0.0001; two-way ANOVA followed by Bonferroni’s *post hoc* test; *P < 0.05, ***P < 0.001), but the proportion of uni/bipolar-shaped cells remains unchanged. On the other hand, treatment with *Bacurd2* siRNAs leads to a significant decrease in the proportion of uni/bipolar-shaped cells and multipolar-shaped cells, with no significant difference in the proportion of round-shaped cells. Scale bar represents 20 μm.

In the course of their radial migration, embryonic cortical cells adopt different modes of migration from the germinal VZ, through to the IZ and the CP [16,17]. Hence, we analysed the morphology of GFP-labelled neurons to describe the cellular basis for the defective migration of cells as a result of perturbations to *Bacurd2*. Within the IZ, we found that forced expression of *Bacurd2* resulted in a significant increase in the proportion of round-shaped cells which have very short processes (or no detectable processes at all), together with a corresponding decrease in multipolar-shaped neurons; while the proportion of uni/bipolar-shaped neurons was not significantly different (Figure 4). On the other hand, knockdown of *Bacurd2* resulted in a significant increase in the proportion of multipolar-shaped neurons and a concomitant decrease in uni/bipolar neurons, while the proportion of round-shaped neurons was not significantly different. Within the CP, we found that forced expression as well as knockdown of *Bacurd2* resulted in an increase in the proportion of round-shaped cells, together with a decrease in the proportions of uni/bipolar-shaped cells. These documented changes in cell morphology upon siRNA-mediated knockdown were corrected by co-delivery of 0.4 μg/μl BACURD2 construct (Additional file 5: Figure S5). Together, these results demonstrate that disruptions to *Bacurd2* alter the morphologies of embryonic neurons, and this effect could underlie their defective migration within the embryonic E17.5 cortex.

**Figure 5:**
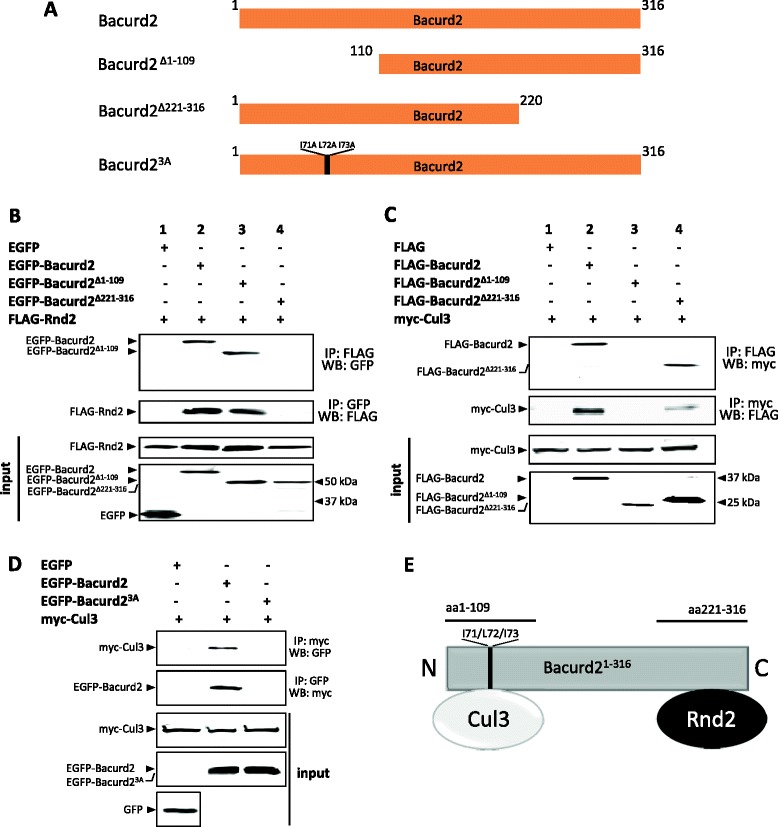
**Bacurd2 interacts with its binding partners through distinct regions of the polypeptide. (A)** Schematic representation of Bacurd2 polypeptide and its mutant variants used in our analysis. **(B)** Reciprocal co-immunoprecipitation assays show that Rnd2 interacts with an N-terminal Bacurd2 truncation mutant but is unable to bind the C-terminal truncation mutant Bacurd2(Δ221-316). **(C)** Cul3 binds Bacurd2(Δ221-316) but not the N-terminal truncation mutant Bacurd2(Δ1-109). **(D)** Mutations to I71/L72/I73 in the Bacurd2(3A) polypeptide sequence disrupt its interaction with Cul3. **(E)** Diagrammatic summary of the interactions between Bacurd2 and its interaction partners Rnd2 and Cul3.

In the following experiments, we wanted to define the interaction domains on Bacurd2 which govern its binding to Rnd2. We cloned truncation mutants of Bacurd2 based on the minimal interaction regions identified in our yeast two-hybrid assay (Additional file 1: Figure S1) and assessed their interaction in co-immunoprecipitation assays using epitope-tagged proteins in heterologous cells (Figure 5). Our results show that while a C-terminal truncation mutant Bacurd2(∆221-316) fails to immunoprecipitate Rnd2, an N-terminal mutant Bacurd2(∆1-109) still interacts with Rnd2 (Figure 5B, lanes 3 to 4). Recently, Bacurd2 was demonstrated to interact with the E3 ubiquitin ligase Cul3 at its N-terminus and signal together to promote fibroblast cell migration *in vitro* [18]. Given that Bacurd2, Rnd2 and Cul3 proteins are all present during mouse brain development (Additional file 2: Figure S2C), we wanted to confirm their protein-protein interaction. As shown, our co-immunoprecipitation experiments reveal that while Cul3 interacts with full-length Bacurd2, as well as a C-terminal truncation mutant, the N-terminal mutant Bacurd2(∆1-109) fails to immunoprecipitate Cul3 (Figure 5C). In addition, we engineered missense mutations I71A/L72A/I73A to Bacurd2 (named as Bacurd2(3A), the location of these amino acids are indicated in bold text on Figure 5A) which are reported to disrupt its BTB domain [18], and we found that this variant did not interact with Cul3 (Figure 5D). Therefore, these studies demonstrate that Bacurd2 interacts with Rnd2 as well as Cul3 via the C- and N-termini, respectively (summarised in Figure 5E).

**Figure 6:**
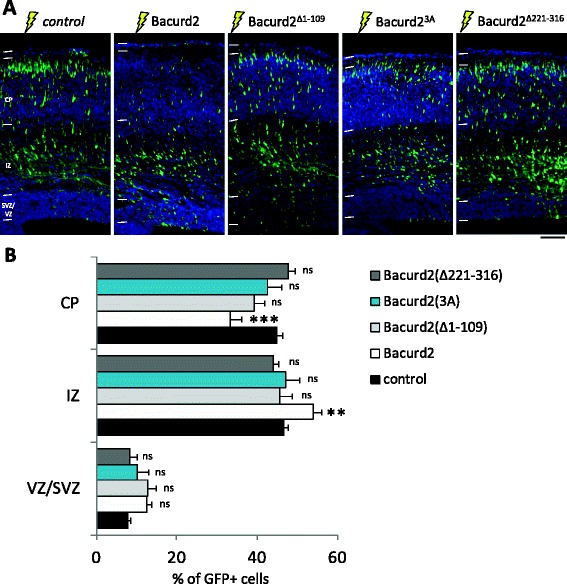
**The effect of forced expression of Bacurd2 and its mutated variants on cell migration within the embryonic E17.5 cortex. (A)** Coronal sections of E17.5 embryonic cortex following E14.5 *in utero* electroporation with a bicistronic construct encoding GFP vector only (control), or together with Bacurd2 or its mutant variants. **(B)** Forced expression of Bacurd2 disrupts the migration of GFP-labelled cells in the embryonic cortex, when compared with control treatment. On the other hand, forced expression of the N-terminal mutants Bacurd2(Δ1-109), Bacurd2(3A) or the C-terminal Bacurd2(Δ221-316) variant did not significantly disrupt the migration of cells (*N* > 4,000 cells counted from four to six brains per condition. Distribution of GFP-labelled cells within the VZ/SVZ, IZ and CP of the E17.5 cortex; *F*_8,96_ = 5.38; two-way ANOVA followed by Bonferroni’s *post hoc* test which compares each column to control; *P < 0.05; ***P < 0.001). Scale bar, 100 μm.

Next, we investigated how the Bacurd2 polypeptide influences neuronal migration by performing *in utero* electroporation assays. Specifically, we asked if forced expression of each of the mutated variants of Bacurd2 (that is Bacurd2(∆1-109), Bacurd2(3A) and Bacurd2(∆221-316)) might affect the migration of E14.5 embryonic cortical cells within the E17.5 cortex in a similar manner to wildtype Bacurd2. As shown in Figure 6, we found that while forced expression of full-length Bacurd2 disrupted the migration of embryonic cortical cells into the CP, forced expression of the N-terminal truncation mutants Bacurd2(∆1-109) or Bacurd2(3A) (both of which fail to interact with Cul3) did not significantly disrupt the migration profile of treated cells when compared with control. Similarly, forced expression of the Rnd2-binding defective mutant Bacurd2(∆221-316) mutant did not significantly disrupt the migration profile of GFP-labelled cells. Therefore, overexpression of all three mutants did not disturb migration and this suggests that an intact, full-length Bacurd2 polypeptide is important for its cell migration functions within the embryonic cortex.

### A Bacurd2:Rnd2 chimeric construct influences radial migration within the embryonic cortex

**Figure 7:**
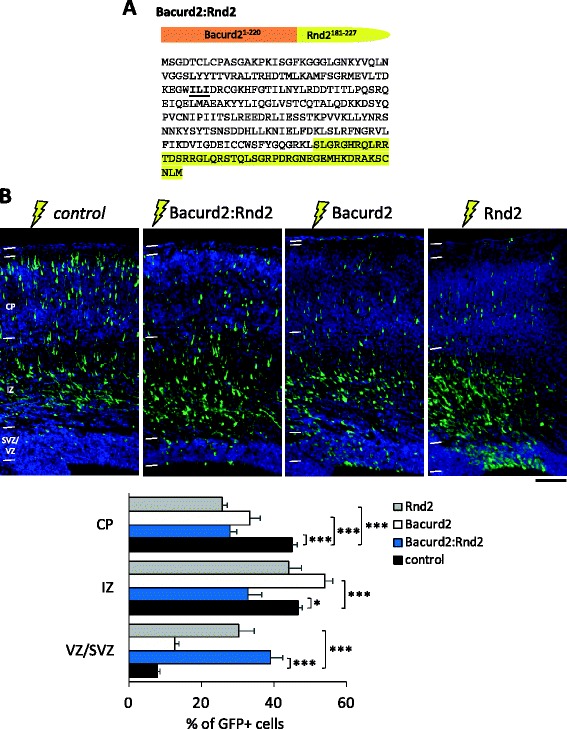
**Forced expression of Bacurd2:Rnd2 impairs radial migration** ***in vivo*** **. (A)** Illustration of the protein resulting comprising the N-terminal region of Bacurd2(1-220) together with the C-terminal region of Rnd2 which mediates the migration of embryonic cortical neurons *in vivo* [7]. **(B)** Forced expression of Bacurd2:Rnd2 impairs cell migration within the embryonic E17.5 cortex, as do cells which overexpress either Bacurd2 or Rnd2 (*N* > 2,400 cells counted from three to four brains per condition; *F*_6,63_ = 55.34; *P* < 0.0001; two-way ANOVA followed by Bonferroni’s *post hoc* test). Scale bar, 100 μm.

Based on our analysis of Bacurd2 and its mutants in migration (Figure 6), we reasoned that the Bacurd2 polypeptide must coordinate cell migration through its protein-protein interactions at its N- and C-termini. To explore the possibility that Bacurd2 might signal cell migration in concert with Rnd2, we designed a polypeptide expression construct comprising a fusion between the N-terminal Bacurd2(aa1-220) sequence together with the C-terminal sequence of Rnd2(aa181-227) (Figure 7A). It was recently discovered that the C-terminal (aa181-227) region of Rnd2 is important for signalling cell migration *in vivo* [7], and so we cloned this region of Rnd2 in place of Bacurd2(aa221-316) to generate a chimeric molecule. When we introduced this construct into E14.5 born cortical cells, we found that forced expression of the Bacurd2:Rnd2 disrupts radial migration in a manner which was distinct to Rnd2 or Bacurd2 overexpression alone (Figure 7B). Notably, we found that forced expression of Bacurd2 led to a significant accumulation of cells in the IZ and a failure of cells to reach the CP, while forced expression of Rnd2 resulted in a significant accumulation of cells in the VZ but not the IZ. In contrast, forced expression of Bacurd2:Rnd2 led to a significant accumulation of cells in the VZ and IZ. Consistent with these distinct effects on cell migration, we found that each different treatment altered the morphology of IZ and CP cells in different ways (Additional file 6: Figure S6).

**Figure 8:**
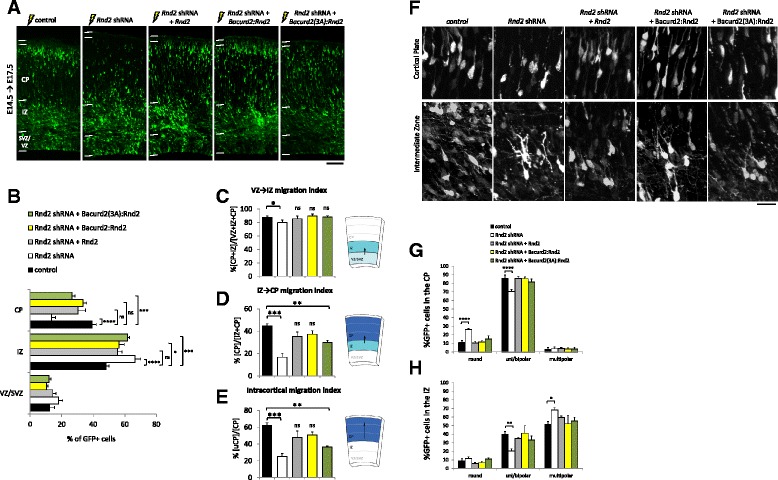
**The defective migration of** ***Rnd2*** **-deficient neurons is restored by co-delivery of Bacurd2:Rnd2. (A,B)** The defective migration of *Rnd2* shRNA is corrected by co-delivery of Rnd2, Bacurd2:Rnd2 and, to a lesser extent, Bacurd2(3A):Rnd2 (*N* > 2,000 cells per condition; *F*_8,39_ = 17.36; *P* < 0.0001; two-way ANOVA by Bonferroni’s *post hoc* test). **(C**-**E)** Cell entry index calculated as the proportion of cells within each subcompartment. *Rnd2* shRNA treatment impairs VZ-to-IZ entry, but is corrected by co-delivery of Rnd2, Bacurd2:Rnd2 and Bacurd2(3A):Rnd2 (*F*_4,13_ = 4.210, *P* = 0.021; one-way ANOVA) (C). Co-delivery of Rnd2 or Bacurd2:Rnd2 restores IZ-to-CP entry of *Rnd2* deficient neurons (*F*_4,13_ = 16.31, *P* < 0.0001; one-way ANOVA) **(D)**, as well as their migration to the upper CP (*N* > 295 cells per condition; *F*_4,13_ = 18.01, *P* < 0.0001; one-way ANOVA) **(E)**. Bacurd2(3A):Rnd2 does not significantly improve IZ-to-CP entry **(D)**, nor the intracortical migration of *Rnd2*-deficient cells **(E). (F)** The morphology of IZ and CP neurons. **(G)** *Rnd2* shRNA-treated CP neurons show an increase in the proportion of round cells and a concomitant reduction in uni/bipolar shaped cells (*N* > 250 cells per condition; *F*_8,57_ = 8.64, *P* < 0.0001; two-way ANOVA followed by Bonferroni’s *post hoc* test). Co-delivery of Rnd2, Bacurd2:Rnd2 or Bacurd2(3A):Rnd2 restores the morphological profile of *Rnd2*shRNA-treated neurons within the CP. **(H)** *Rnd2* shRNA-treated IZ neurons show a significant increase in the proportion of multipolar cells and a concomitant reduction in uni/bipolar shaped cells (*N* > 640 cells per condition; *F*_8,51_ = 3.497, *P* = 0.0027; two-way ANOVA followed by Bonferroni’s *post hoc* test). Co-delivery of Rnd2, Bacurd2:Rnd2 and Bacurd2(3A):Rnd2 restored the morphologies of *Rnd2* shRNA-treated IZ neurons to control profile. Data was collected from three to four independent brains per condition. Scale bar represents 100 μm **(A)** and 20 μm **(F)**.

It was reported that suppression of *Rnd2* by RNAi significantly disrupted cell migration within the embryonic E17.5 cortex, including their multipolar-to-bipolar transition from the IZ to the CP [6,7,10]. Hence, we wanted to determine if the migration defect of *Rnd2*-deficient cells could be restored by modulating Bacurd2 signalling. We began with control experiments to confirm that the defective migration of *Rnd2* shRNA-treated cells could be corrected by co-delivering an expression construct encoding *Rnd2* which is not targeted by the shRNA vector (Figure 8A,B) [6,7]. Next, we asked whether forced expression of full-length Bacurd2 could compensate for the defective migration of *Rnd2* shRNA-treated cells, but we did not observe a restoration of cell migration in our assay (*n* = 6 brains per condition, data not shown). In contrast, co-delivery of Bacurd2:Rnd2 significantly improved the migration of *Rnd2* shRNA-treated cells (Figure 8A), with cells reaching the cortical plate at levels not significantly different to control treatment (Figure 8B; 39.37% ± 2.57% of cells within the CP of control samples versus 33.45% ± 2.4% of *Rnd2* shRNA+ Bacurd2:Rnd2 treated cortices; *F*_8,39_ = 17.36; *P* < 0.0001; two-way ANOVA; *post hoc t*-test *P* > 0.05). In addition to this result, we were also interested to determine whether I71A/L72A/I73A substitution mutations to the BTB domain of Bacurd2 which disrupt its binding to Cul3 were relevant to its cell migration functions. Thus, we performed parallel rescue experiments to co-deliver Bacurd2(3A):Rnd2 (which is defective in Cul3 binding; see Additional file 7: Figure S7) together with *Rnd2* shRNA in embryonic E14.5 cortical cells. Our results show that while treatment with Bacurd2(3A):Rnd2 improved the migration of *Rnd2* shRNA-treated cells, the proportion of GFP-labelled cells within the CP remained significantly decreased compared with control condition (Figure 8A,B; 13.43% ± 2.76% of cells within the CP of *Rnd2*shRNA-treated cortices versus 26.38% ± 2.06% in *Rnd2*shRNA+ Bacurd2(3A):Rnd2 treated cortices versus 39.37% ± 2.57% of cells within the CP of control samples; *F*_8,39_ = 17.36; *P* < 0.0001; two-way ANOVA; *post hoc t*-test ***P < 0.0001).

We previously demonstrated that Rnd2 controls the morphological transitions undertaken by migrating neurons as they reach the CP, including their multipolar-to-bipolar transition as they leave the IZ and enter the CP [6,7]. Thus, we analysed the migration index of GFP-labelled cells in our current rescue experiments to understand how neurons enter the IZ (Figure 8C) and the CP (Figure 8D,E). As a control experiment, we first confirmed that *Rnd2*-deficient cells are defective in their migration from the VZ to the IZ and CP in a cell autonomous fashion, as previously reported [6,7] (Figure 8C,D,E). We then observed that the IZ migration defect of *Rnd2* shRNA-treated cells is restored with either the Bacurd2:Rnd2 or Bacurd2(3A):Rnd2 to levels which are not significantly different to control profile (Figure 8C). In contrast, the defective CP-entry of *Rnd2*-deficient cells was efficiently restored only when Bacurd2:Rnd2 was co-delivered, but not with Bacurd2(3A):Rnd2 (Figure 8D). Furthermore, we found that the defective intracortical distribution of *Rnd2*-deficient cells was only corrected by co-delivery of Bacurd2:Rnd2, but not with Bacurd2(3A):Rnd2 (Figure 8E).

Finally, we analysed GFP-labelled neurons within the IZ and CP to determine whether the abnormal morphologies of *Rnd2* shRNA-treated neurons could be corrected by co-delivery of Bacurd2:Rnd2. We first investigated the morphologies of neurons within the IZ of *Rnd2* shRNA electroporated brains and found a significant increase in the proportion of multipolar-shaped neurons compared with control treatment, a result which is consistent with our previous reports describing failed multipolar-to-bipolar transition of *Rnd2*-deficient cells [6-8,10,11] (Figure 8F). Also, we observed that co-delivery of *Rnd2* restores the morphologies of *Rnd2* shRNA-treated neurons to a distribution which is not significantly different to control treatment. In contrast, we found that the morphological profiles of *Rnd2* shRNA + Bacurd2:Rnd2 treated cells within the IZ and CP were restored to a profile resembling control condition, as were *Rnd2*shRNA cells co-treated with Bacurd2(3A):Rnd2 (Figure 8G,H). Taken together, our results collectively demonstrate that Bacurd2 coordinates cell migration within the embryonic cortex and influences the morphological transitions of immature neurons as they transit through the IZ, as well as when their radial distribution within the CP. Despite the caveat that our Bacurd2:Rnd2 chimeric construct represents an artificial model of a Bacurd2-Rnd2 signal transducer, our results suggest that Bacurd2 and Rnd2 promote cell migration within the embryonic cortex.

## Discussion

We previously reported that Rnd2 regulates the migration of newborn embryonic cortical neurons [6,7]; hence, we wanted to clarify the downstream signalling pathway through which Rnd2 modulates this activity. In this study, we have identified Bacurd2 as an interacting partner to Rnd2 which influences radial migration during cerebral cortex development. Notably, both Rnd2 and RhoA signalling are crucial to radial migration [6,7,19], and Bacurd2 suppresses RhoA to influence cell migration *in vitro* [18]. Therefore, we were motivated to characterise the neuronal functions for Bacurd2 within the embryonic cortex. We find that disruptions to *Bacurd2* impair cell migration and alter the multipolar-to-bipolar transition of embryonic cortical neurons. Thus, our study introduces Bacurd2 as a new player in neuronal development.

The ability for immature neurons to migrate is a function which is sensitive to Rnd2 levels, with too much or too little disrupting this process [6,7,10]. Given the role for Bacurd2 in targeting RhoA for degradation by the Cul3 ubiquitin ligase complex, it is possible that Bacurd2 may act as a substrate adaptor for the degradation of Rnd proteins. As such, Bacurd2 could target Rnd2 for degradation via the Cul3 ubiquitin ligase complex so as to promote radial migration. However, the role for Bacurd2 in radial migration is also likely to be mediated through RhoA regulation as well. In the future, it will be important to determine the relative contributions of both of these postulated signalling mechanisms for Bacurd2 which influence the development of cerebral cortical neurons.

In our functional studies, we found that truncation of the C-terminal region of Bacurd2 abolishes its binding to Rnd2 and disrupts its cell migration functions in embryonic cortical cells. Furthermore, a truncation of the N-terminal region of Bacurd2 or the introduction of missense mutations I71A/L72A/I73A within its BTB-domain (both of which disrupts its binding to Cul3) similarly abolishes its effects on cell migration *in vivo*. From these findings, we surmise that the ability for the Bacurd2 polypeptide to control cell migration relies on its N- and C-terminal domains. With the knowledge that Cul3 and Rnd2 interact with the C- and N- termini of Bacurd2, respectively, together with our evidence that these proteins are detected throughout the course of brain development, our future studies will address their combined roles within the context of neurodifferentiation.

A significant challenge in our study was to address how Bacurd2 and Rnd2 might signal to promote cell migration. We took a drastic approach by tethering a Rnd2(aa181-227) polypeptide sequence to the C-terminal (aa221-316) region of Bacurd2. We found that Bacurd2:Rnd2 could restore the defective migration of *Rnd2*shRNA-treated neurons within the embryonic cortex. But, how can we explain the basis for this rescue? One possible explanation relates to the migration-promoting functions of Rnd2 and Bacurd2. In the case of Rnd2, it was reported that its aa181-227 C-terminal region is important for subcellular localisation and promotion of activity within the perinuclear region of immature neurons to signal migration [7,20]. On the other hand, studies with a triple I71A/L72A/I73A point mutation variant of Bacurd2(3A) protein show an importance for Cul3 binding to signal cell migration in a wound-healing assay [18] as well as embryonic cell migration (this study). When we attempted to reconcile these findings through our own experimentation, our results show that Bacurd2:Rnd2 restores the migration of *Rnd2* shRNA-treated neurons. While the roles for both Bacurd2 and Rnd2 in neuronal development remain to be better understood, it will be important to determine if these molecules are important for the terminal differentiation of cortical neurons, including their dendritic arborisation and synaptic connectivity. More broadly, given the roles for Bacurd and Rnd proteins in the regulation of the early steps of neurogenesis [7,12,21,22], a better understanding of their combined signalling activities will reveal their specific contributions to the development and function of cerebral cortical neurons in health and disease.

## Conclusions

The molecular regulation of radial migration is complex and involves multiple signalling factors which promote directional movement as well as neurite outgrowth as immature neurons position themselves within the developing cerebral cortex. We have identified Bacurd2 as a new player which promotes the migration of immature cortical projection neurons in a concentration-sensitive manner. In addition, we have characterised Bacurd2 as a novel interacting partner to Rnd2, a known regulator of radial migration within the embryonic cortex. Our discovery supports the notion that Rnd2 interacts with specific binding partners (such as Bacurd2) to control neuronal migration during cortical development.

## Methods

*Animals* - Mice were housed, bred and treated within the animal facilities at Monash University. All animal procedures are approved by the Animal Ethics Committee within Monash University (Licenses MARP/08-104 and MARP/2012/068) and are compliant with guidelines provided by the National Health and Medical Research Council of Australia.

*Cloning, plasmids and siRNA* - cDNA for murine Bacurd2 (also known as Tnfaip1) was amplified from Invitrogen clone accession number BC003906 using primers, 5′-CATCATCAATTGatgtcaggggacacctgtctg 3′ (forward) and 5′-CATCATCAATTGtcagtc acgatgagtggactg-3′ (reverse) (Invitrogen, Grand Island, USA). Truncation mutants were constructed by the standard PCR cloning strategy or DNA synthesis of the entire cDNA (Life Technologies, Carlsbad, USA). Complementary DNA (cDNA) fragments were cloned into the EcoRI site of pEGFP-C2 (Clontech, Mountain View, USA) and pCIG2-Flag. Expression constructs for Rnd2 and Rnd3 were previously described [2]. The Bacurd2:Rnd2 cDNA encodes the following polypeptide which is engineered as a fusion between aa1-220 of Bacurd2 and aa181-227 of Rnd2: MSGDTCLCPASGAKPKISGFKGGGLGNKYVQLNVGGSLYYTTVRALTRHDTMLKAMFSGRMEVLTDKEGW**ILI**DRCGKHFGTILNYLRDDTITLPQSRQEIQELMAEAKYYLIQGLVSTCQTALQDKKDSYQPVCNIPIITSLREEDRLIESSTKPVVKLLYNRSNNKYSYTSNSDDHLLKNIELFDKLSLRFNGRVLFIKDVIGDEICCWSFYGQGRKL*SLGRGHRQLRRTDSRRGLQRSTQLSGRPDRGNEGEMHKDRAKSCNLM* (the Rnd2 C-terminal polypeptide sequence is represented in italics, while the amino acids in bold are targeted for alanine substitution in the Bacurd2(3A):Rnd2 construct). All constructs were sequenced verified and plasmids produced using PureYield™ Midi-prep kits (Promega, Madison, USA).

*Immuno-precipitation and Western blotting* - Six embryonic 14.5 mice brains were homogenised (ProScientific Pro200 homogenizer, ProScientific, Oxford, USA) in ice-cold lysis buffer (20 mM Tris (pH 7.5), 150 mM NaCl, 1% IGEL-PAL and 0.1%SDS supplemented with protease inhibitor). The resultant lysate was sonicated then centrifuged at 10,000 × *g* for 20 min at 4°C before transferring to a fresh tube. The cleared lysate was then incubated with antibodies: mouse IgG (Millipore, Billerica, USA) and mouse anti Bacurd2 (Abmart, Shanghai, China) overnight at 4°C with rotation. The solution was then incubated with protein A sepharose beads for 2 h at 4°C and then washed three times with the cold protein lysis buffer. Immunoprecipitated proteins were eluted with loading buffer containing 50 mM DTT and samples were analysed on a 10% SDS page gel. Following membrane transfer, the membranes were incubated with anti-Rnd2 antibodies, followed by secondary goat-anti-rabbit antibody (Li-Cor IRDye 680 LT). Signals were detected with Odyssey® infrared imaging system (Li-Cor 9201-02, Lincoln USA) for analysis. Native antibodies to Bacurd2 (epitope is SPSEDEDTFE Abmart) and Rnd2 (sc-33543; Santa Cruz Biotechnologies, Santa Cruz, USA) were used.

*In utero electroporation* - *In utero* electroporation experiments are performed as described [23]. High-quality, low endotoxin plasmid preparations (Qiagen, Limburg, The Netherlands) of DNA vectors were injected at 1 μg/μl for each plasmid, together with Fast Green (0.05%, Sigma, St. Louis, USA). For RNAi experiments, Dharmacon siRNA targeting pools for Bacurd2 were injected at 10 μM concentration together with GFP expression plasmid at 1 μg/μl concentration. Following recovery from *in utero* electroporation, the mice were sacrificed by cervical dislocation, and the embryonic brains were harvested by dissection in cold PBS and preserved for tissue processing, cryosectioning (16 μm) and fluorescence immunostaining. Images of brain sections were captured on an epifluorescence microscope (Olympus, Tokyo, Japan) equipped with a CCD camera (SPOT, Sterling Heights, USA). Subdivisions of the embryonic cortex (VZ/SVZ, IZ and CP) were identified based on cell density as visualised with 4′6-diamidino-2-phenylindole (DAPI) staining. Cell counting was performed blind to the condition on representative fields of sections of electroporated brains using ImageJ software.

## Additional files

Additional file 1: Figure S1. Identification of Bacurd2 as a binding partner to Rnd2 in a yeast two-hybrid assay. Representation of results of a yeast two-hybrid interaction screen using an Rnd2 bait which lacks its C-terminal (CAAX) motif. **(A)** Identification of multiple cDNA prey encoding Bacurd2 polypeptide. Complementation assays confirm association of Bacurd2 preys with Rnd2 bait. **(B)** Growth of yeast transfected with Rnd2 bait and Bacurd2 prey construct on nutritional selection medium (lacking histidine and adenine). Double-transfected yeast harbouring Bacurd2 prey and pLaminC bait, or Bacurd2 prey with p53 bait do not grow on this selection medium. Additional file 2: Figure S2. Immunodetection of Bacurd2 and Rnd2. **(A)** Western blotting of HEK293T cell lysates transfected with FLAG-Bacurd2 construct and immunoblotted with our mouse monoclonal antibody. A specific signal of approximately 37 kDa is detected, which corresponds to the FLAG-Bacurd2 signal in an immunoblot with FLAG antibody. **(B)** Western blotting of HEK293T cell lysates transfected with FLAG-Rnd2 construct and immunoblotted with a rabbit polyclonal antibody (Santa Cruz Biotechnologies, Santa Cruz, USA). A specific signal of approximately 25 kDa is detected and which corresponds to the FLAG-Rnd2 signal in an immunoblot with FLAG antibody. **(C)** Western blotting using antibodies to Bacurd2, Rnd2 and Cul3 on mouse brain lysates harvested from the indicated timepoints during mouse brain development. The signal for β-actin was used as a loading control. **(D-E)** Fluorescence immunostaining of E14.5 embryonic cortex using preimmune serum (D) or Bacurd2 antibody (E, green signal). Nuclei were counterstained with DAPI. Scale bar, 50 μm. Additional file 3: Figure S3. Perturbations to *Bacurd2* do not disturb neuronal differentiation. **(A)** GFP-labelled cells which co-express Tuj1 (arrow) or do not co-express Tuj1 (arrowheads) are identified in a representative section of E17.5 embryonic cortex. **(B)** Quantification studies of the proportion of GFP-labelled cells which co-express the neuronal marker Tuj1 (*N* > 1,700 cells counted from at least three brains per condition; one-way ANOVA followed by Bonferroni’s *post hoc t*-test) reveal no significant difference in GFP+/Tuj1+ cells following overexpression or knockdown of Bacurd2 (*F*_2,13_ = 1.6; *P* = 0.24; *P* > 0.5). **(C)** Two-way ANOVA analysis of GFP+/Tuj1− cells in each cortical subcompartment reveals no significant difference in their distribution following overexpression or knockdown of Bacurd2 (*F*_4,39_ = 0.44; *P* = 0.77; *P* > 0.5). Graph plots mean ± SEM. Scale bar, 50 μm. Additional file 4: Figure S4. The defective migration of *Bacurd2* siRNA-treated cells is augmented by co-delivery of human BACURD2. **(A)** To ask if co-treatment with 0.4 μg/μl or 1.0 μg/μl of BACURD2 construct restores their migration of siRNA-treated cells, we performed two-way ANOVA followed by Bonferroni’s multiple comparisons test. We found that treatment with either concentrations of BACURD2 improved the migration of cortical cells (*N* > 1,450 cells counted per condition, *F*_6,45_ = 15, *P* < 0.0001). **(B)** An analysis of their intracortical distribution (that is within the lower, medial and upper CP) reveals that the defective migration of Bacurd2 siRNA-treated cells is restored with co-delivery of 0.4 μg/μl of BACURD2 construct, but treatment with 1.0 μg/μl of BACURD2 did not restore their positioning within the upper CP (*N* > 500 cells per condition, *F*_6,42_ = 15.27, *P* < 0.0001; two-way ANOVA followed by Bonferroni’s *post hoc* test). Graph plots mean ± SEM. Scale bar represents 100 μm. Additional file 5: Figure S5.
*Bacurd2* controls cell shape *in vivo*. **(A)** The morphology of neurons within the CP and the IZ in representative brain sections electroporated with control (GFP only) vector, *Bacurd2* siRNAs or *Bacurd2* siRNAs with the indicated concentrations of BACURD2 expression vector. Arrowheads point to round-shaped cells. **(B)** Within the CP, knockdown of *Bacurd2* leads to a significant increase in the proportion of round cells and a decrease in uni/bipolar-shaped cells (*N* > 300 cells counted from three brains per condition; *F*_6,60_ = 24.60; *P* < 0.0001; two-way ANOVA followed by Bonferroni’s *post hoc* test; **P* < 0.05, ****P* < 0.001). These effects on cell shape are corrected with co-delivery of 0.4 μg/μl of BACURD2 construct. **(C)** Within the IZ, treatment with *Bacurd2* siRNAs leads to a significant decrease in the proportion of uni/bipolar-shaped cells and multipolar-shaped cells, with no significant difference in the proportion of round-shaped cells when compared with control. (*N* > 500 cells counted from three brains per condition; *F*_6,48_ = 22.52; *P* < 0.0001; two-way ANOVA followed by Bonferroni’s *post hoc* test; **P* < 0.05, ****P* < 0.001). Co-delivery of either 0.4 μg/μl of BACURD2 construct or 1.0 μg/μl of BACURD2 construct corrects these *Bacurd2* siRNA-induced changes to cell shape within the IZ. Scale bar represents 20 μm. Additional file 6: Figure S6. Cell shape profiles following treatment with *Bacurd2:Rnd2* **,** ***Bacurd2*** **or** ***Rnd2*** **.** Analysis of the morphologies of IZ and CP neurons following forced expression of Bacurd2:Rnd2, Bacurd2 or Rnd2. There was an interaction between treatment and distribution of cell shapes in the CP (*N* > 190 cells from three to four brains per condition; *F*_6,60_ = 8.332, *P* < 0.0001; two-way ANOVA followed by Bonferroni’s *post hoc* test) **(A)** and cells within the IZ (*N* > 390 cells from three to four brains per condition; *F*_6,51_ = 7.537, *P* < 0.0001; two-way ANOVA followed by Bonferroni’s *post hoc* test) **(B)**. Graph plots mean ± SEM. Additional file 7: Figure S7. Point mutations I71A/L72A/I73A alter binding to Cul3. **(A)**, Diagrammatic representation of Bacurd2 full-length polypeptide sequence, along with Bacurd2:Rnd2 contruct, and a Bacurd2(3A):Rnd2. Yellow indicates the sequence of Rnd2 polypeptide fused to the C-terminal fragment of Bacurd2^1-220^ polypeptide (see the ‘Methods’ section for polypeptide sequence). Bold letters identify substitution mutations I71A/L72A/I73A within the BTB-domain of the Bacurd2 polypeptide fragment which mediates Cul3 binding. **(B)** Reciprocal co-imunoprecipitation experiments were performed to confirm that the Bacurd2:Rnd2 interacts with Cul3, but not with Bacurd2(3A):Rnd2. Input lanes confirm the presence of all proteins evaluated in this experiment.

## Abbreviations

bHLH: basic helix-loop-helix
CP: cortical plate
E: embryonic day
EGFP: enhanced green fluorescent protein
IZ: intermediate zone
lCP: lower cortical plate
mCP: medial cortical plate
siRNA: small inhibitory RNA
uCP: upper cortical plate
VZ: ventricular zone
