# Supplementary material for: Bacurd2 is a novel interacting partner to Rnd2 which controls radial migration within the developing mammalian cerebral cortex
Source: Neural Dev. 2015 Mar 31;10:9. doi: 10.1186/s13064-015-0032-z (PMC4433056; doi:10.1186/s13064-015-0032-z)
Supplement: Additional file 1: Figure S1. — Identification of Bacurd2 as a binding partner to Rnd2 in a yeast two-hybrid assay. Representation of results of a yeast two-hybrid interaction screen using an Rnd2 bait which lacks its C-terminal (CAAX) motif. (A) Identification of multiple cDNA prey encoding Bacurd2 polypeptide. Complementation assays confirm association of Bacurd2 preys with Rnd2 bait. (B) Growth of yeast transfected with Rnd2 bait and Bacurd2 prey construct on nutritional selection medium (lacking histidine and adenine). Double-transfected yeast harbouring Bacurd2 prey and pLaminC bait, or Bacurd2 prey with p53 bait do not grow on this selection medium. [file 13064_2015_32_MOESM1_ESM.pdf]

A

Bait	Prey	Growth in media - *his*, -*ade*
Rnd2$^{1-223}$	Bacurd2 $^{1-316}$	+++
Rnd2$^{1-223}$	Bacurd2 $^{242-316}$	+++
pLaminC	Bacurd2 $^{242-316}$	-
p53	Bacurd2 $^{242-316}$	-

B

Rnd2$^{1-223}$ bait

pLaminC bait

p53 bait

Bacurd2$^{1-316}$ prey

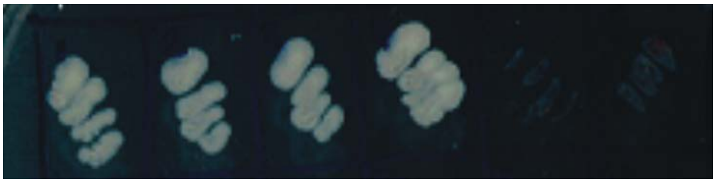

Growth in media -his, -ade
