# Supplementary figures and images for: Bacurd2 is a novel interacting partner to Rnd2 which controls radial migration within the developing mammalian cerebral cortex
Source: Neural Dev. 2015 Mar 31;10:9. doi: 10.1186/s13064-015-0032-z (PMC4433056; doi:10.1186/s13064-015-0032-z)

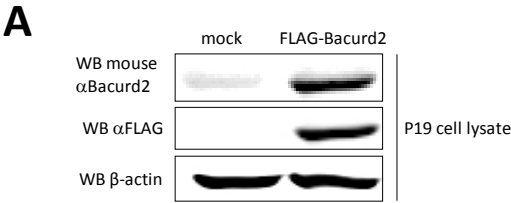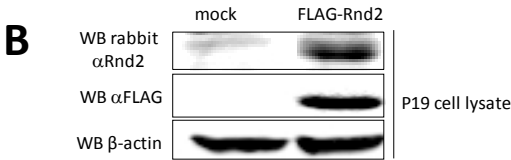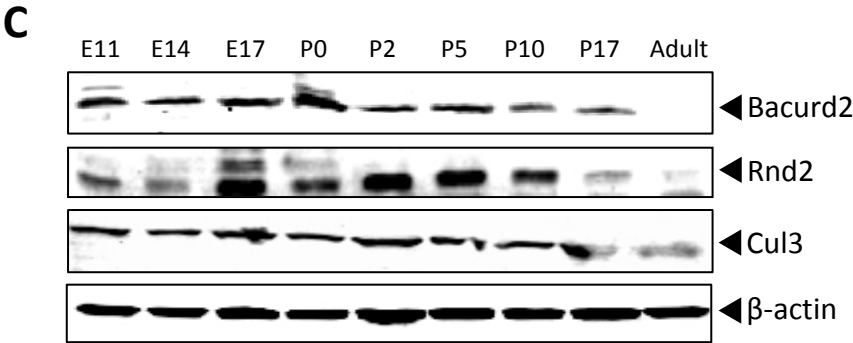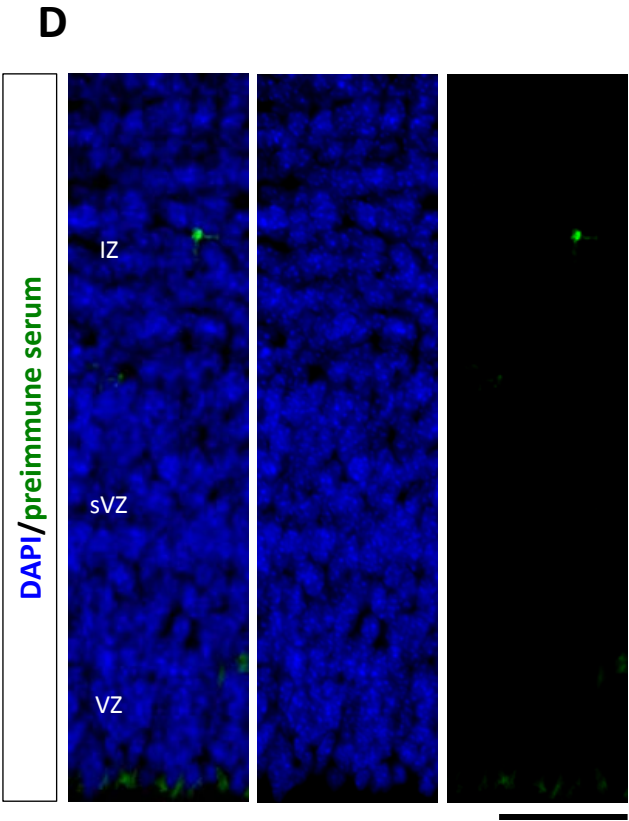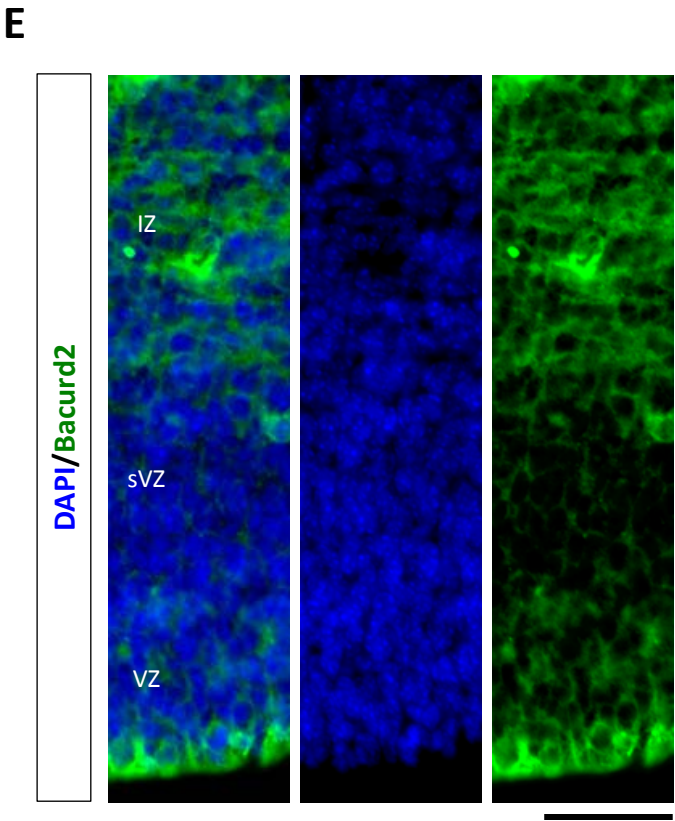

Supplement: Additional file 2: Figure S2. — Immunodetection of Bacurd2 and Rnd2. (A) Western blotting of HEK293T cell lysates transfected with FLAG-Bacurd2 construct and immunoblotted with our mouse monoclonal antibody. A specific signal of approximately 37 kDa is detected, which corresponds to the FLAG-Bacurd2 signal in an immunoblot with FLAG antibody. (B) Western blotting of HEK293T cell lysates transfected with FLAG-Rnd2 construct and immunoblotted with a rabbit polyclonal antibody (Santa Cruz Biotechnologies, Santa Cruz, USA). A specific signal of approximately 25 kDa is detected and which corresponds to the FLAG-Rnd2 signal in an immunoblot with FLAG antibody. (C) Western blotting using antibodies to Bacurd2, Rnd2 and Cul3 on mouse brain lysates harvested from the indicated timepoints during mouse brain development. The signal for β-actin was used as a loading control. (D-E) Fluorescence immunostaining of E14.5 embryonic cortex using preimmune serum (D) or Bacurd2 antibody (E, green signal). Nuclei were counterstained with DAPI. Scale bar, 50 μm. [file 13064_2015_32_MOESM2_ESM.pdf]

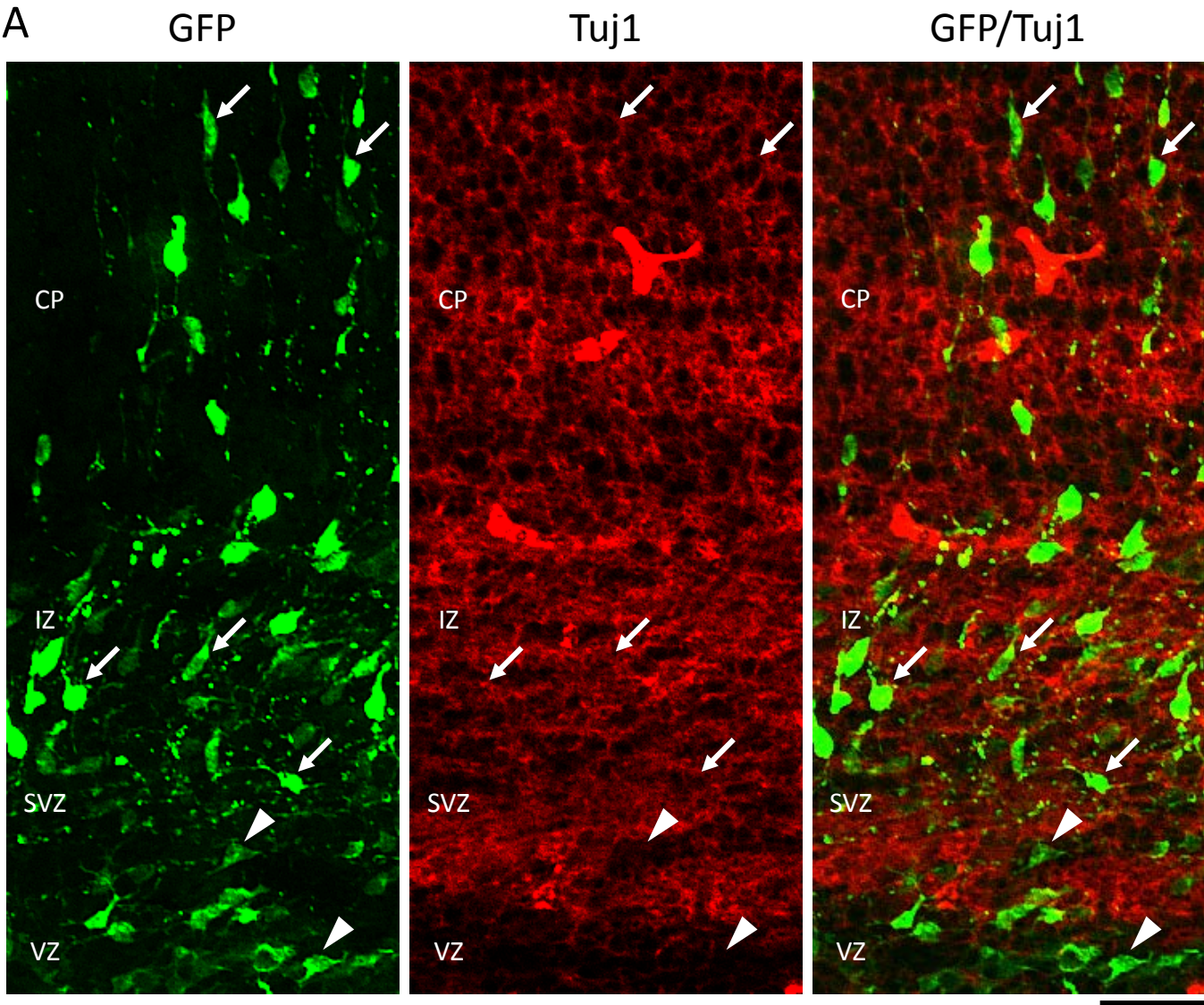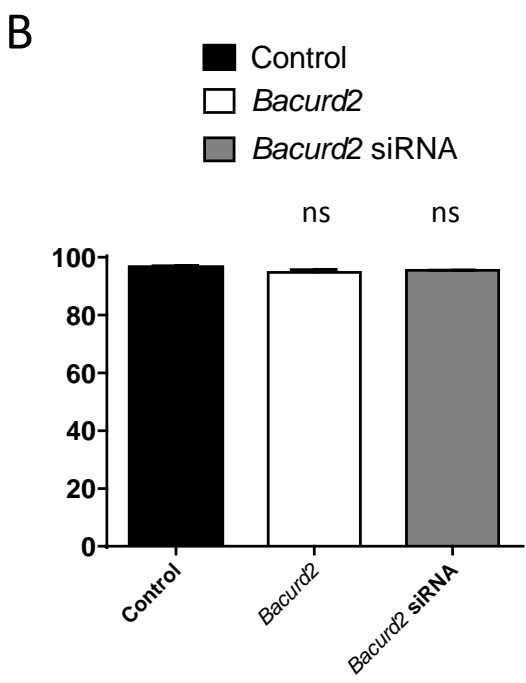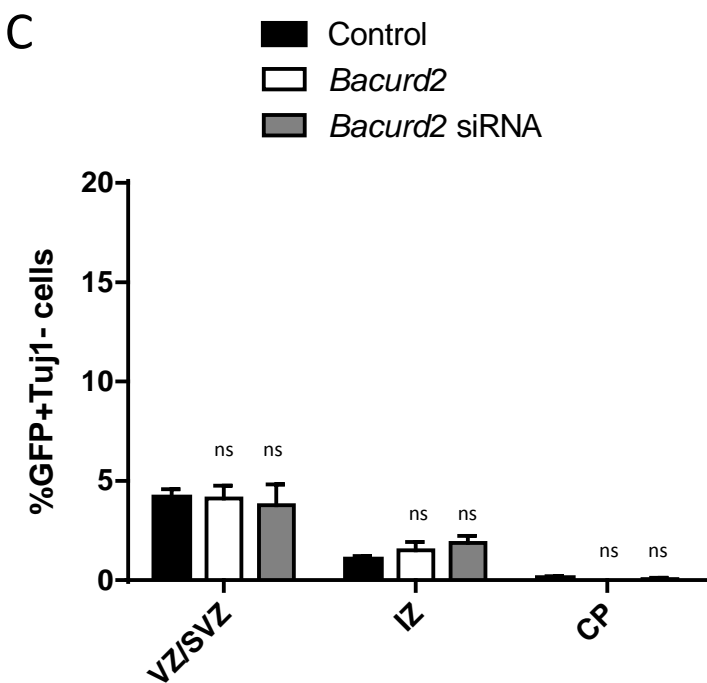

Supplement: Additional file 3: Figure S3. — Perturbations to Bacurd2 do not disturb neuronal differentiation. (A) GFP-labelled cells which co-express Tuj1 (arrow) or do not co-express Tuj1 (arrowheads) are identified in a representative section of E17.5 embryonic cortex. (B) Quantification studies of the proportion of GFP-labelled cells which co-express the neuronal marker Tuj1 (N > 1,700 cells counted from at least three brains per condition; one-way ANOVA followed by Bonferroni’s post hoc t-test) reveal no significant difference in GFP+/Tuj1+ cells following overexpression or knockdown of Bacurd2 (F 2,13 = 1.6; P = 0.24; P > 0.5). (C) Two-way ANOVA analysis of GFP+/Tuj1− cells in each cortical subcompartment reveals no significant difference in their distribution following overexpression or knockdown of Bacurd2 (F 4,39 = 0.44; P = 0.77; P > 0.5). Graph plots mean ± SEM. Scale bar, 50 μm. [file 13064_2015_32_MOESM3_ESM.pdf]

SUPPLEMENTARY FIG 4

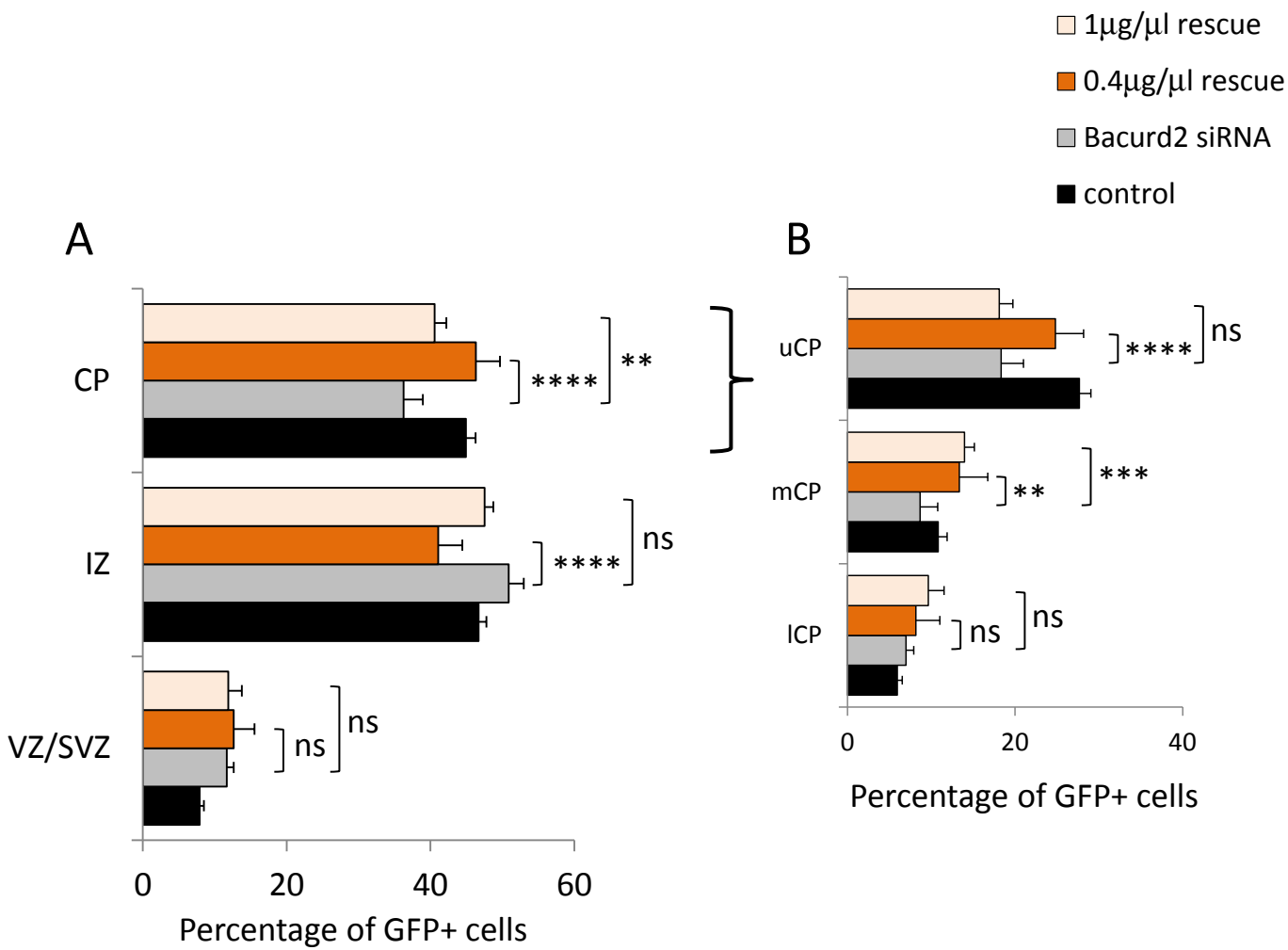

Supplement: Additional file 4: Figure S4. — The defective migration of Bacurd2 siRNA-treated cells is augmented by co-delivery of human BACURD2. (A) To ask if co-treatment with 0.4 μg/μl or 1.0 μg/μl of BACURD2 construct restores their migration of siRNA-treated cells, we performed two-way ANOVA followed by Bonferroni’s multiple comparisons test. We found that treatment with either concentrations of BACURD2 improved the migration of cortical cells (N > 1,450 cells counted per condition, F 6,45 = 15, P < 0.0001). (B) An analysis of their intracortical distribution (that is within the lower, medial and upper CP) reveals that the defective migration of Bacurd2 siRNA-treated cells is restored with co-delivery of 0.4 μg/μl of BACURD2 construct, but treatment with 1.0 μg/μl of BACURD2 did not restore their positioning within the upper CP (N > 500 cells per condition, F 6,42 = 15.27, P < 0.0001; two-way ANOVA followed by Bonferroni’s post hoc test). Graph plots mean ± SEM. Scale bar represents 100 μm. [file 13064_2015_32_MOESM4_ESM.pdf]

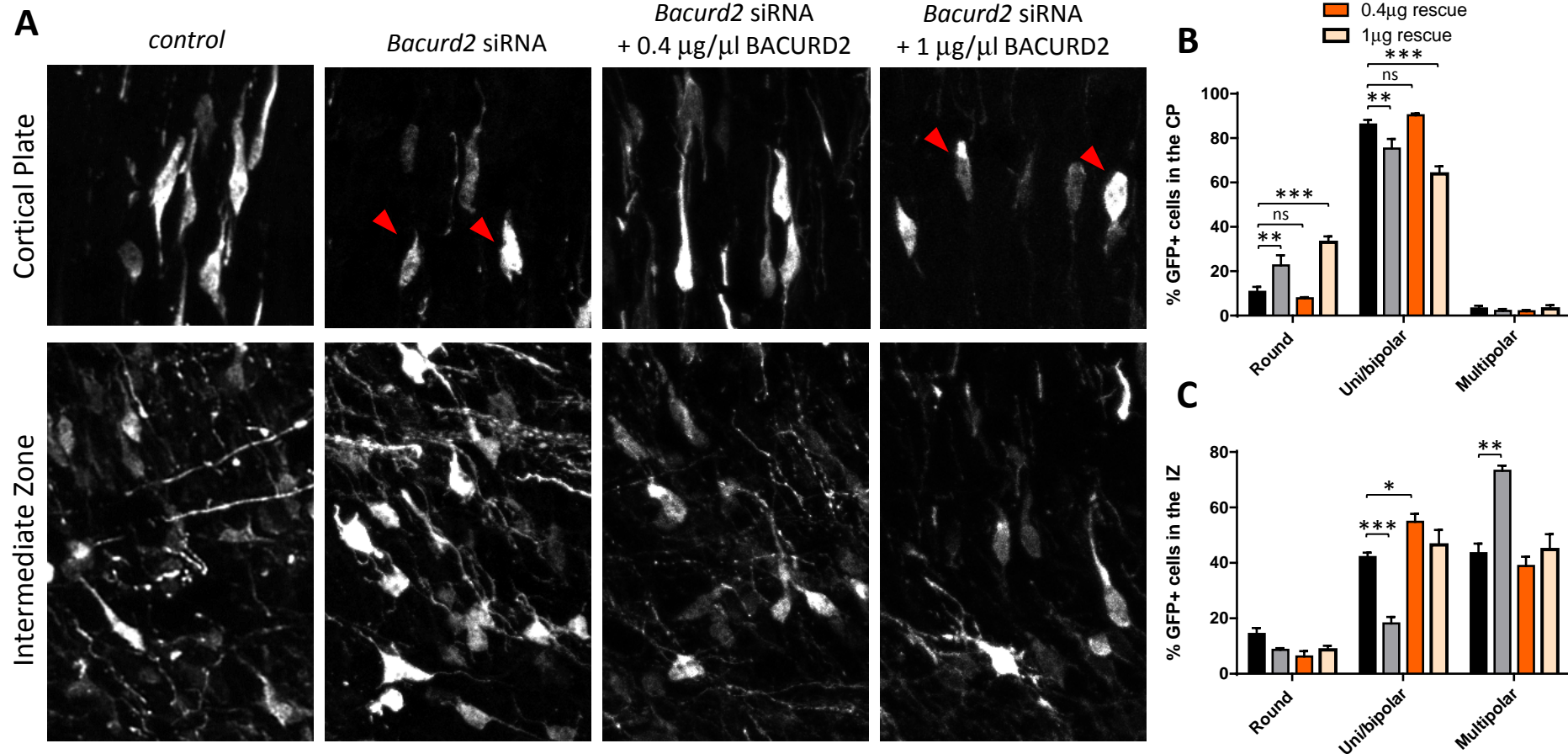

Supplement: Additional file 5: Figure S5. — Bacurd2 controls cell shape in vivo. (A) The morphology of neurons within the CP and the IZ in representative brain sections electroporated with control (GFP only) vector, Bacurd2 siRNAs or Bacurd2 siRNAs with the indicated concentrations of BACURD2 expression vector. Arrowheads point to round-shaped cells. (B) Within the CP, knockdown of Bacurd2 leads to a significant increase in the proportion of round cells and a decrease in uni/bipolar-shaped cells (N > 300 cells counted from three brains per condition; F 6,60 = 24.60; P < 0.0001; two-way ANOVA followed by Bonferroni’s post hoc test; *P < 0.05, ***P < 0.001). These effects on cell shape are corrected with co-delivery of 0.4 μg/μl of BACURD2 construct. (C) Within the IZ, treatment with Bacurd2 siRNAs leads to a significant decrease in the proportion of uni/bipolar-shaped cells and multipolar-shaped cells, with no significant difference in the proportion of round-shaped cells when compared with control. (N > 500 cells counted from three brains per condition; F 6,48 = 22.52; P < 0.0001; two-way ANOVA followed by Bonferroni’s post hoc test; *P < 0.05, ***P < 0.001). Co-delivery of either 0.4 μg/μl of BACURD2 construct or 1.0 μg/μl of BACURD2 construct corrects these Bacurd2 siRNA-induced changes to cell shape within the IZ. Scale bar represents 20 μm. [file 13064_2015_32_MOESM5_ESM.pdf]

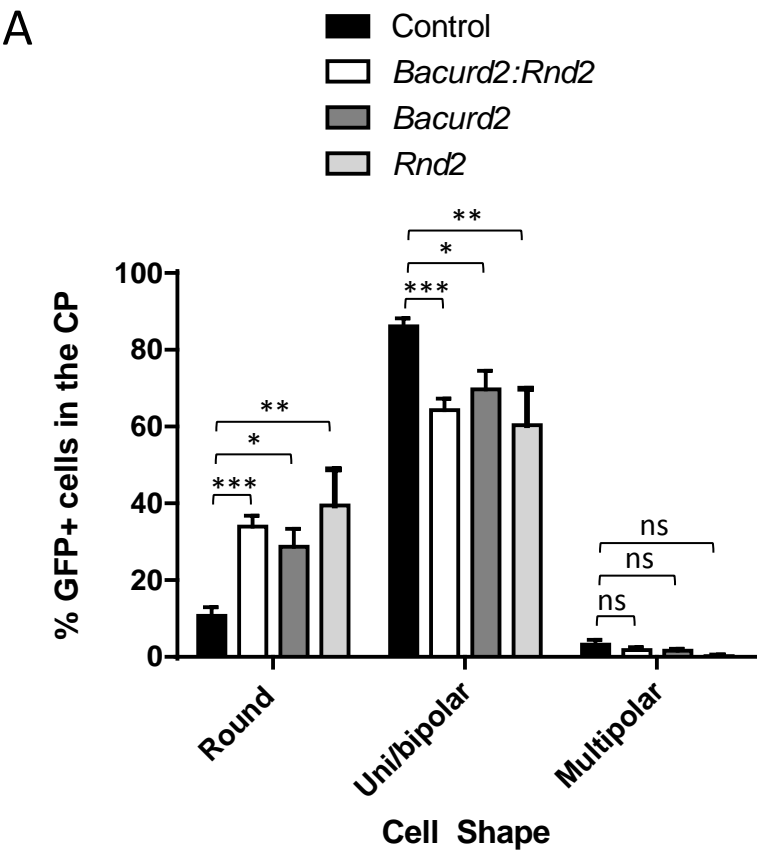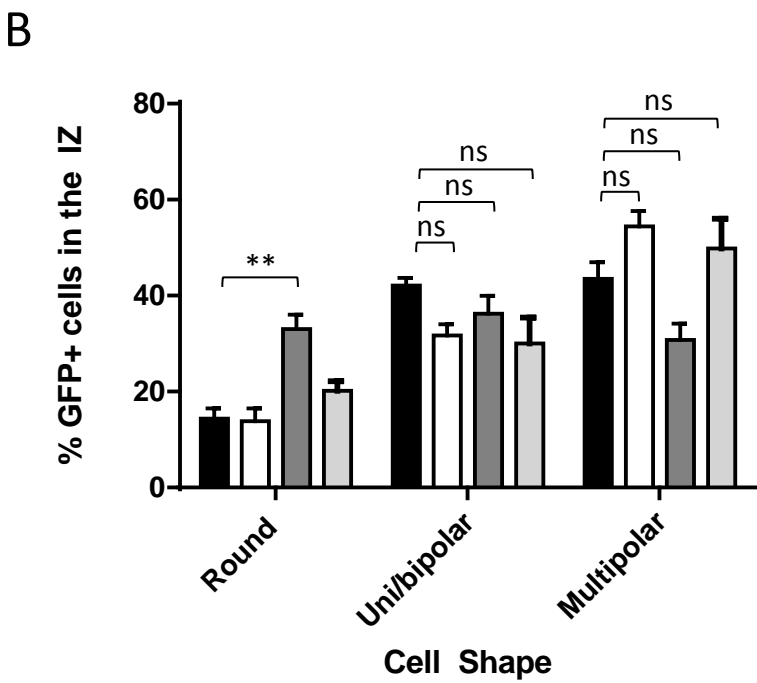

Supplement: Additional file 6: Figure S6. — Cell shape profiles following treatment with Bacurd2:Rnd2 , Bacurd2 or Rnd2 . Analysis of the morphologies of IZ and CP neurons following forced expression of Bacurd2:Rnd2, Bacurd2 or Rnd2. There was an interaction between treatment and distribution of cell shapes in the CP (N > 190 cells from three to four brains per condition; F 6,60 = 8.332, P < 0.0001; two-way ANOVA followed by Bonferroni’s post hoc test) (A) and cells within the IZ (N > 390 cells from three to four brains per condition; F 6,51 = 7.537, P < 0.0001; two-way ANOVA followed by Bonferroni’s post hoc test) (B). Graph plots mean ± SEM. [file 13064_2015_32_MOESM6_ESM.pdf]
