# Supplementary material for: Bacurd2 is a novel interacting partner to Rnd2 which controls radial migration within the developing mammalian cerebral cortex
Source: Neural Dev. 2015 Mar 31;10:9. doi: 10.1186/s13064-015-0032-z (PMC4433056; doi:10.1186/s13064-015-0032-z)

SUPPLEMENTARY FIG 7

A

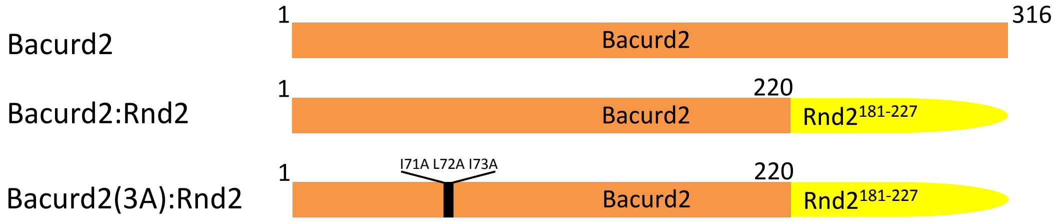

Bacurd2:Rnd2

MSGDTCLCPASGAKPKISGFKGGGLGNKYVQLNVGGSLYYTTVRALTRHDTMLKAMFSG
RMEVLTDKEGW**ILI**DRCGKHFGTILNYLRDDTITLPQSRQEIQELMAEAKYYLIQGLVS
TCQTALQDKKDSYQPVCNIPIITSLREEDRLIESSTKPVVKLLYNRSNNKYSYTSNSDD
HLLKNIELFDKLSLRFNGRVLFIKDVIGDEICCWSFYGQGRKLSLGRGHRQLRRTDSRR
GLQRSTQLSGRPDRGNEGEMHKDRAKSCNLM

Bacurd2(3A):Rnd2

MSGDTCLCPASGAKPKISGFKGGGLGNKYVQLNVGGSLYYTTVRALTRHDTMLKAMFSG
RMEVLTDKEGW**AAA**DRCGKHFGTILNYLRDDTITLPQSRQEIQELMAEAKYYLIQGLVS
TCQTALQDKKDSYQPVCNIPIITSLREEDRLIESSTKPVVKLLYNRSNNKYSYTSNSDD
HLLKNIELFDKLSLRFNGRVLFIKDVIGDEICCWSFYGQGRKLSLGRGHRQLRRTDSRR
GLQRSTQLSGRPDRGNEGEMHKDRAKSCNLM

B

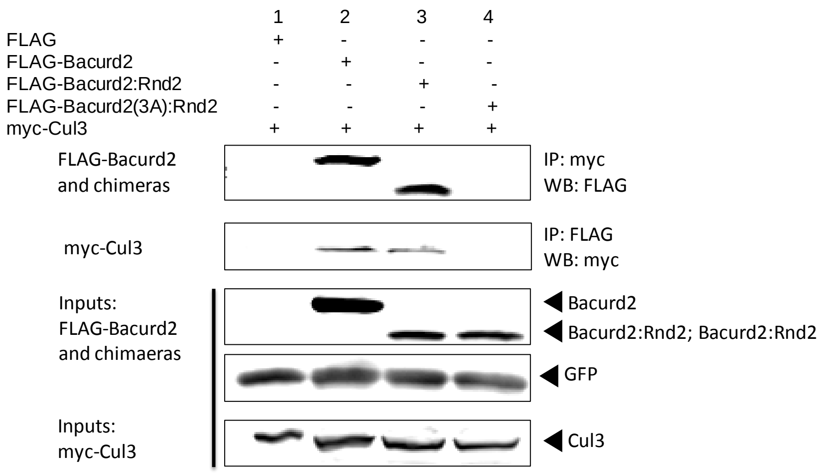

Supplement: Additional file 7: Figure S7. — Point mutations I71A/L72A/I73A alter binding to Cul3. (A), Diagrammatic representation of Bacurd2 full-length polypeptide sequence, along with Bacurd2:Rnd2 contruct, and a Bacurd2(3A):Rnd2. Yellow indicates the sequence of Rnd2 polypeptide fused to the C-terminal fragment of Bacurd21-220 polypeptide (see the ‘Methods’ section for polypeptide sequence). Bold letters identify substitution mutations I71A/L72A/I73A within the BTB-domain of the Bacurd2 polypeptide fragment which mediates Cul3 binding. (B) Reciprocal co-imunoprecipitation experiments were performed to confirm that the Bacurd2:Rnd2 interacts with Cul3, but not with Bacurd2(3A):Rnd2. Input lanes confirm the presence of all proteins evaluated in this experiment. [file 13064_2015_32_MOESM7_ESM.pdf]
